# Experimental study for inorganic and organic profiling of toy makeup products: Estimating the potential threat to child health

**DOI:** 10.1007/s11356-024-33362-2

**Published:** 2024-05-02

**Authors:** Selda Mercan, Mihriban Dilan Kilic, Simge Zengin, Murat Yayla

**Affiliations:** Institute of Forensic Sciences and Legal Medicine, Department of Science, Istanbul University- Cerrahpasa, 34500 Buyukcekmece, Istanbul, Turkey

**Keywords:** Children, Cosmetic toys, Allergen, Inorganic element, Exposure

## Abstract

Inorganic elements are added to toys as impurities to give desired stability, brightness, flexibility, and color; however, these elements may cause numerous health issues after acute or chronic exposure. In this study, the inorganic profile of 14 elements (Al, As, Ba, Cd, Co, Cr, Cu, Hg, Mn, Ni, Se, Sb, Pb, and Zn) in 63 toy makeup products was identified by inductively coupled plasma-mass spectrometry after microwave acid digestion method. Additionally, organic allergen fragrance was investigated by gas chromatography-mass spectrometry. The systemic exposure dosage (SED), margin of safety (MoS), lifetime cancer risk (LCR), hazard quotient (HQ), and hazard indices were used to assess the safety evaluation. Then, 57 out of 63 samples (90.48%) exceeded the limits at least for one toxic element with descending order Ni > Cr > Co > Pb > Sb > Cd > As > Hg. The SED values were compared with tolerable daily intake values and remarkably differences were found for Al and Pb. The MoS values for 57.15% of samples exceeded the limit value for Al, As, Cd, Co, Hg, Mn, Sb, and Zn elements. The LCR values were observed at 100% (*n* = 63), 79.37% (*n* = 50), 85.71% (*n* = 54), 77.78% (*n* = 49), and 18.87% (*n* = 10) for Cr, Ni, As, Pb, and Cd, respectively. Also, the skin sensitization risks were obtained for Cr and Ni at 26.980% (*n* = 17) and 9.52% (*n* = 6), respectively. The HQ values for 80% of samples were found to be ≥ 1 at least for one parameter. The investigation of fragrance allergens in samples did not show any significant ingredients. As a result, toy makeup products marketed in local stores were found to be predominantly unsafe. Children should be protected from harmful chemicals by regular monitoring and strict measures.

**Supplementary Information:**

The online version contains supplementary material available at 10.1007/s11356-024-33362-2.

## Introduction

Toys are items frequently used by children under 14 years of age for learning, improving skills, or enjoying. However, toys are also potential sources of toxic chemicals (e.g., inorganic elements, allergens, and carcinogens) when produced improperly and may cause various adverse effects in the case of prolonged exposure. Children are commonly exposed to these toxic chemicals, which are involved in contaminated toys, unwittingly through mouthing, ingestion, inhalation, dermal, and eye contact (Ter Burg et al. 2008; Safavi et al. 2019; Van Engelen et al. 2008).

Recently, with the growing popularity of the makeup toy industry, toy makeup which comprises eye shadow, nail polish, and lipstick has been widely used by children and could be contaminated by toxic elements during manufacturing. Children are more vulnerable to these toxic elements due to inadequate metabolism, immature detoxification system, greater absorption rate, and consuming more foods per unit body weight than adults and, thus, are generally considered separately in risk assessments (D. e. S. Gul et al. 2022; Landrigan et al. 2004). Furthermore, toy makeup products are applied to the child’s thinnest and weak body parts such as the face (5 mm) and eye (2 mm) and stay on the skin for a long time as adult cosmetics (Brandão & Gontijo 2012; Corazza et al. 2009; Temesvári et al. 2009).

Inorganic elements (e.g., antimony (Sb), arsenic (As), cadmium (Cd), chromium (Cr), cobalt (Co), copper (Cu), lead (Pb), manganese (Mn), mercury (Hg), nickel (Ni), and zinc (Zn)) are involved in various matrices such as toy makeup products, cosmetics, paints, jewelry, green coffees, and biological specimens and can be accumulated in the body over time after exposure (Mercan et al. 2015, 2022, 2023; Mercan 2020; Şemen et al. 2017). These elements are added to toys intentionally to enhance softness, flexibility, brightness, and color as well as extend shelf life or unintentionally ways such as leakage of the inorganic element from the instrument, the addition of low-quality chemicals, and usage of toxic element-contaminated water (Godoi et al. 2009; D. e. S. Gul et al. 2022; Guney & Zagury 2012; Negev et al. 2018; Sadighara et al. 2023).

Numerous regulatory agencies have announced the regulated maximum permissible limits for certain toxic elements (As, Cd, Cr, Co, Hg, Ni, Sb, and Pb) involved in adult cosmetics as impurities to avoid over-exposure (Bund 2016; Health Canada 2012; FDA 2022; Rathod & Desai 2021; Turkish Medicine and Medical Devices Agency 2005).

On the other hand, the fragrance ingredients, among toxic chemicals, are also added to the toy makeup to avoid unpleasant odors or to increase their appeal to consumers; however, they are potentially allergenic which can trigger respiratory diseases (e.g., asthma and rhinitis), neurotoxicity, endocrine disruption, and contact dermatitis in children (Masuck et al. 2010; Wang et al. 2018; Wolkoff & Nielsen 2017). Since the concentration and composition of common fragrance allergens in toys is increasingly coming to public attention (Chen et al. 2022), the European Union (EU) has set requirements for fragrance allergens in the Directive 2009/48/EC of the European Parliament on the Safety of Toys and EN71-13:2014 (European 2009; NSAI 2014).

Studies have been conducted on children’s toys (D. e. S. Gul et al. 2022; Kang & Zhu 2015; Karas & Frankowski 2018; Negev et al. 2018; Yazdanfar et al. 2022), jewelry (A. Gul et al. 2023; Hillyer et al. 2014; Yost & Weidenhamer 2008), finger paints, watercolors, acrylic paints, crayons, (M. R. Khan et al. 2021; Mračević et al. 2022), and face paints (Rebelo et al. 2015; Salles et al. 2023). There are also studies on allergens (Lv et al. 2013; Masuck et al. 2010, 2011; Rastogi et al. 1999; Wang et al. 2018), as well as organic chemicals (Guo et al. 2018; Szczepańska et al. 2018) used in toys. However, there are limited studies investigating organic and/or inorganic ingredients of toy makeup products simultaneously (Corazza et al. 2009; Rastogi 1992; Rastogi et al. 1999). To the best of our knowledge, there is no comprehensive study carried out evaluating the safety of toy makeup including both toxic and essential inorganics by calculating health risks besides allergens up to date.

From this perspective, this study is aimed at monitoring the contents of inorganic elements and allergenic fragrances inside the toy makeup in the market and comparing results with maximum permissible limits. The profiling of 14 inorganic elements as well as allergen fragrance inside of the toy makeup were monitored for the first time with this study. Also, the safety of each product was evaluated to assess possible health risks for children through margin of safety (MoS), systemic exposure dosage (SED), lifetime cancer risk (LCR), hazard quotient (HQ), and hazard index (HI) expressions.

## Material and methods

### Chemicals and equipment

All the reagents and chemicals used in the inorganic analysis were of analytical grade. Nitric acid 65% (v/v) solution was purchased from Merck Suprapur® (Merck, Darmstadt, Germany) for sample preparation. The certificated calibration solution mixture (multi-element, 10 mg mL^−1^) and mono-element standards (Hg; 1000 mg mL^−1^) were obtained from High-Purity Standards (Charleston, SC) to achieve a calibration curve. As internal standards (ISs), Indium (In) and Gallium (Ga) (1000 mg mL^−1^) were purchased (Absolute Standards, Inc., Hamden, CT, USA). Two different certificated reference materials (CRM) were used ((i.) the organic reach soil BCR-700 (Institute for Reference Materials and Measurements, Belgium) and (ii.) the light sandy soil as 7002 (Czech Metrology Institute, Czech Republic)) for obtaining benchmark information for the analytical method. The stability of the ICP-MS system was monitored by a tuning solution (10 µg mL^−1^) (High-Purity Standards, Charleston, SC). High-purity argon gas (> 99.999% purity, Habas, Turkey) was used to attain the argon plasma.

During the inorganic profiling, the samples were digested by a microwave digestion system (with temperature and pressure sensors) (CEM Mars 5, Matthews, NC, USA, 2008). A Thermo Scientific X Series-II Inductively Coupled Plasma-Mass Spectrometry (ICP-MS) (Thermo Fisher Scientific, Bremen, Germany, 2008) and a CETAC, ASX 520 (Omaha, Neb., USA) auto-sampler were used for elemental analysis. To supply ultra-pure water (18.2 MΩ cm), a Millipore Direct-Q®3 UV purification system (Millipore, Molsheim, France) was used.

All the reagents and chemicals used in the organic analysis were of analytical grade. The methanol was obtained from Merck Suprapur® (Merck, Darmstadt, Germany) as an organic solvent, and anhydrous sodium sulfate (99%) was purchased from Sigma-Aldrich (Steinheim, Germany) to remove water.

Gas chromatography-mass spectrometry (GC–MS) analyses carried out for organic profiling were performed by an HP 6890 series gas chromatography coupled with an HP 5975B series mass selective detector (MSD) (Agilent, Santa Clara, California, USA). Automated sampling was made by HP 7683B (Agilent Technologies, Palo Alto, CA USA), and Agilent Technologies, HP-5MS capillary GC column (30 m × 250 µm i.d. × 0.25 µm film thickness) was used in this study.

### Preparation of standards and calibration

The first part of the experimental studies was inorganic analysis, and the analytical method used for the determination of inorganic elements was validated by quality control parameters including linearity, sensitivity, accuracy, and recovery to ensure the reliability of the method. The measurement of linearity was conducted at an 11-point calibration curve for Al, As, Ba, Cd, Co, Cr, Cu, Hg, Mn, Ni, Se, Sb, and Pb which ranged from 0.1 to 150 ng mL^−1^ and 12-point calibration curve for Zn, where incremental amounts ranged from 1 to 400 ng mL^−1^. Ga and In (ISs) (20 ng mL^−1^ of) were added to all calibration solutions and samples. Since there are no certified makeup products, the accuracy of the method was monitored by using two soil CRMs. The sensitivity was estimated by determining detection limits for all monitored analytes. Three and ten times the standard deviation, obtained from the results of blank solutions (*n* = 10), were determined as the limit of detection (LOD) and limit of quantification (LOQ), respectively. As the lack of inorganic element-free cosmetic products, the recovery of the analytical method was evaluated with the standard addition method by adding 10 and 20 ng mL^−1^ concentrations of standard solutions to the microwave vessels, and three independent replicates were prepared for each spike level. To prevent possible contamination, all disposable tubes and digestion vessels were soaked into 10% HNO_3_ solutions before the sample preparation step.

In the second part, screening of organic substances for the determination of allergen fragrance in samples was carried out qualitatively by GC–MS. Due to the lack of reference standards for allergen fragrances, these substances could not be quantified in this study, and also, validation parameters (LOD, LOQ, linearity, and recovery) could not be studied.

### Sample collection and preparation

**Fig. 1:**
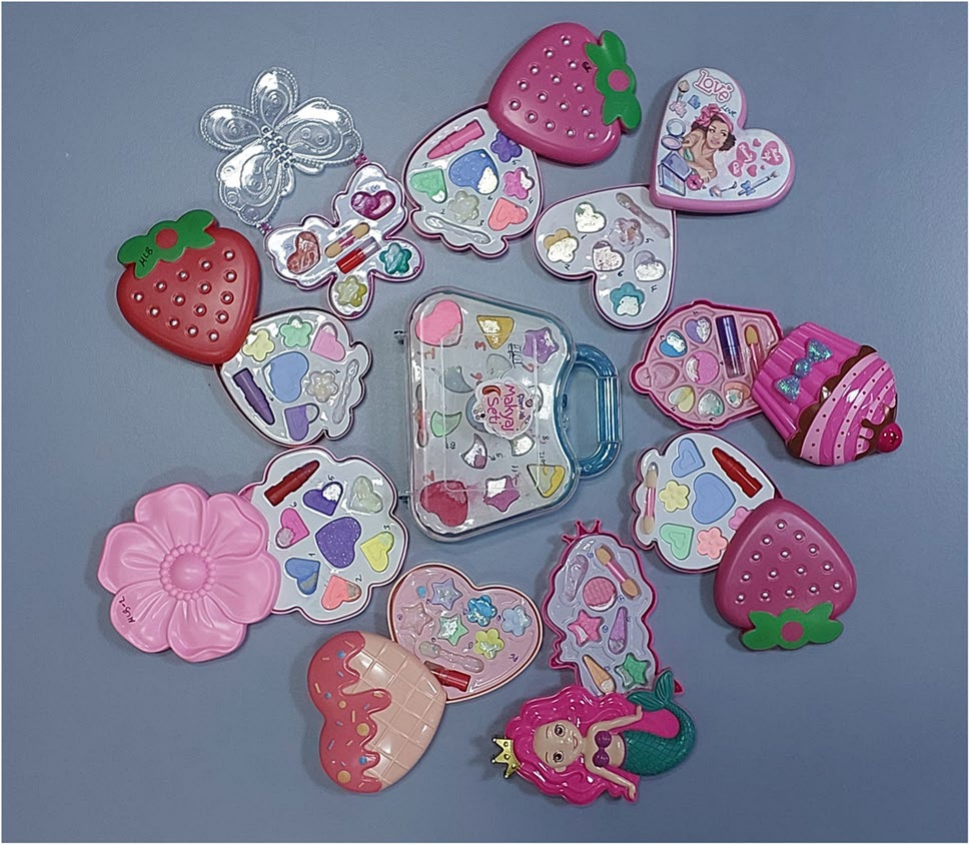
Toy makeup products analyzed in this study (10 different brands)

A total of 63 toy makeup products representing 10 different brands were purchased from toy stores in Istanbul (in Fig. 1**)**, and each brand was coded as BRAND A, B, C, D, E, F, G, H, I, and J. Of these products, 51 toy makeups are originated from Turkey (8 of 10 brands) and the rest of the products (*n* = 12) are imported from China.

#### Sample preparation for inorganic analysis

The sample preparation steps were carried out by following Mercan et al.’s (Mercan et al. 2023) recent paper, which was adopted for adult cosmetic products. Samples were dried at 37 °C in the oven before sieving to provide homogenization and stored at room temperature in polyethylene bottles until the analysis by ICP-MS.

#### Sample preparation for organic analysis

The samples were prepared by using Bawazir et al. (2023) and Lu et al. (2021) papers with slight modifications. Then, 0.25 ± 0.05 g of each sample was weighted into 50 mL volumetric flasks; then, 5 mL of methanol and 0.5 g of anhydrous sodium sulfate were added to the flasks (Bawazir et al. 2023; Lu et al. 2021). The capped flask was intensely vortexed until achieving homogenous suspension and sonicated for 10 min in an ultrasonic bath at 50 °C with 250 W ultrasonic power. The samples were centrifuged for 5 min at 5000 rpm, and the supernatants were transferred into different volumetric flasks before filtration with a cellulose acetate syringe filter (0.22 µm). Then, 200 µL organic content was taken from each sample, reconstituted with methanol at the final volume of 500 µL, and injected into the GC–MS system for qualitative analysis. All experiments were carried out in triplicate throughout the study.

### Analysis conditions for inorganic and organic analyses

The instrumental operating conditions of the ICP-MS system were obtained as follows: plasma gas flow was 13 L min^−1^, nebulizer gas flow was set at 0.87 L min^−1^, radio frequency power was 1400 W, the auxiliary gas flow was 0.8 L min^−1^, 100 sweeps/replicate were used, spray chamber temperature was set at 3 °C, sample uptake was 45 s, and dwell time was 0.01 s. To ensure quality control and reveal possible carryover, ISs-added blank samples were monitored in every batch of analysis, and CRMs were analyzed after every ten samples.

The operating conditions of the GC–MS system were recorded as follows: carrier gas was helium at a flow rate of 1 mL min^−1^, and injection port was held at 250 °C. The temperature gradient started at 50 °C (held for 2 min) and raised with 15 °C min^−1^ to reach 265 °C. The final temperature was held for 12 min, and the total run time was 28 min for comprehensive scanning. Samples were analyzed in split mode (5:1), and transfer line and ionization source temperatures were set at 280 and 230 °C, respectively. The mass detector was operated in the electron impact (EI) mode with 70 eV and in full scan mode (*m/z* 50–600). Total ion chromatograms were evaluated to determine the analytes. For the identification of the allergen fragrance ingredients, the MS library was used.

### Health risk assessment

Health risks of toxic elements present in cosmetic products can be computed by the *margin of safety (MoS)*, which is the ratio of the no observed adverse effect level (NOAEL) of the product to the *systemic exposure dosage (SED)* as shown in Eq. 1 (SCCS 2018). Based on the Turkish Medicine and Medical Devices Agency, MoS value is acceptable up to 150 and considered safe for 5-year-old children (Turkish Medicine and Medical Devices Agency 2005).

1 $$\mathrm{MoS }= \frac{{\text{NOAEL}}}{{\text{SED}}}$$

NOAEL refers to a level of exposure where no adverse effect is observed and is calculated based on dermal reference doses (RfDs) using the following expression in Eq. 2:

2 $${\text{NOAEL}}=\mathrm{ RfD }\times \mathrm{UF }\times {\text{MF}}$$

where UF is the uncertainty factor, and MF is the modifying factor. The default values for MF and UF are 1 and 100, respectively. RfDs represent dermal reference doses of different metals, and values for Al, Cr, Mn, Co, Ni, Cu, Zn, As, Se, Cd, Sb, Ba, Hg, and Pb are 1, 0.0195, 0.00096, 0.0003, 0.02, 0.04, 0.3, 0.0003, 0.005, 0.0000125, 0.00006, 14, 0.0003, and 0.04 mg kg^−1^ day^−1^, respectively (Shomar & Rashkeev 2021).

The safety evaluation of toy makeup products was assessed by systemic exposure dosage (SED), which is the estimation of dermal absorption of metal and computed by the formula given in Eq. 3 (SCCS 2012).

3 $$\mathrm{SED }= \frac{C \times \mathrm{AA }\times {\text{SSA}} \times F \times \mathrm{RF }\times {\text{BF}}}{{\text{BW}}}\mathrm{ x CF}$$

where *C* is the concentration of metal in the toy makeup product (mg kg^−1^), and AA is the amount of cosmetic product applied per day: 0.02 for eye shadows (SCCS 2012); SSA (in cm^2^) represents skin surface area onto which the products are applied and BW depicts the body weight (kg). The SSA/BW ratio is 1.5 for 5 aged children according to the Turkish Medicine and Medical Devices Agency (Turkish Medicine and Medical Devices Agency, 2014). RF is the retention factor: 1.0 for leave-on cosmetic products; *F* is the frequency of application per day, 2; BF is the bioaccessibility factor, 100%; CF is the unit conversion factor, 10^−3^. The values of AA and RF used in the current study are the standard values generated by the Scientific Committee on Consumer Safety (SCCS) (SCCS 2012).

*Lifetime cancer risk (LCR)* is usually calculated for carcinogenic metals. In the current study, LCR was determined by using Eq. 4 (SCCS 2012).

4 $$LCR=\mathrm{ SED }\times {\text{SF}}$$

where SF represents the carcinogenicity slope factors (mg^−1^ kg d), and it estimates the cancer risk per unit intake dose of an agent to cause cancer over an average lifetime. The reported slope factors for Cr, Ni, As, Cd, and Pb are 0.5, 0.91, 1.5, 6.7, and 0.0085 mg^−1^ kg d, respectively (Lim et al. 2018).

*Hazard quotient (HQ)*, which is the ratio of SED of a substance to the RfD of each inorganic element, and hazard index (HI), which is the summation of total HQs of each element were computed by following Eqs. 5 and 6, respectively (El-Aziz et al. 2017).

5 $${\text{HQ}}= \frac{{\text{SED}}}{{\text{RfD}}}$$

6 $${\text{HI}}=\sum {\text{HQ}}$$

Apart from the abovementioned aspects,* the worst-case exposure scenario* is another risk assessment approach, which takes into account different exposure scenario levels. In this study, the worst-case scenario of the 95th percentile was computed as follows (Hajrić et al., 2022); (i) the mean concentrations of each inorganic element for each brand were sorted by ascending order; (ii) the multiplication of the number of samples for each brand with 0.95 occurrence factor, which equals to the “n”; (iii) the *n*^th^ value of ascended concentrations of inorganic elements corresponds to the worst-case scenario of the 95th percentile. Then, these results were compared with reported tolerable daily intake (TDI) values for inorganic elements (Van Engelen et al. 2008).

## Results and discussion

### Method performance of ICP-MS

The linearity, with ≥ 0.999 correlation coefficient (*r*^2^), was found in the ranges of 0.1–150 ng mL^−1^ for Al, As, Ba, Cd, Co, Cr, Cu, Mn, Ni, Se, Sb, and Pb, 1.0–400 ng mL^−1^ and 0.5–20 ng mL^−1^ for Zn and Hg, respectively. The calibration curves displayed good linearity between the response of the instrument and solutions containing elements with a satisfactory *r*^2^ (≥ 0.999). According to the current study, LOQs were obtained between 0.11 and 0.99 ng mL^−1^ for trace elements (Ba, Co, Cr, Cu, Mn, Se, and Zn) and 0.10 and 1.29 ng mL^−1^ for toxic elements (Al, As, Cd, Hg, Ni, Sb, and Pb), while LOD was obtained between 0.03 and 0.30 ng mL^−1^ for trace elements and 0.03 and 0.39 ng mL^−1^ for toxic elements.

The accuracy results monitored by using two soil CRMs were successfully found in the range of 71.77–112.21% for As, Ba, Cd, Co, Cr, Cu, Hg, Mn, Ni, Pb, and Zn in both soil CRMs. The recovery results, obtained from spiked samples, varied from 90.00% (Ni) to 110.17% (Ba) and 93.00% (Ni) to 106.17% (Sb) for 10 ng mL^−1^ and 20 ng mL^−1^ concentrations, respectively. Also, 110.00% and 89.00% for Hg at 5 and 10 ng mL^−1^ concentrations all meet the quality requirements (80.00–120.00%). The relative standard deviations (RSD%) of each sample set (*n* = 3) were found below 16.64%, which indicates the acceptable ranges (< 20.00%). The results of performance parameters confirmed that the developed method was suitable for inorganic element analysis in toy makeups.

### Inorganic profile of toy makeup products

Table 1 represents the concentrations of 14 inorganic elements in 10 different toy makeup brands, while Table S1 shows the concentrations of 8 toxic elements (As, Cd, Cr, Co, Hg, Ni, Sb, and Pb) for each toy makeup sample in detail.

**Table 1:** The min–max, mean, median, and standard deviation (SD) values of 14 elements in 10 toy makeup brands (µg g^−1^)

Element (µg g^−1^)		Al	Cr	Mn	Co	Ni	Cu	Zn	As	Se	Cd	Sb	Ba	Hg	Pb
BRAND A (*n* = 11)	**Min–max**	296**–**11,188	0.65 − 3.54	11.86**–**258.64	0.13**–**1.10	< LOQ**–**1.55	0.67**–**164.68	27.34**–**2223	0.13**–**27.68	< LOQ**–**2.36	< LOQ**–**0.51	< LOQ**–**1.62	1.96**–**222.24	< LOQ	0.37**–**171.20
	**Mean**	6206	2.32	151.24	0.62	0.36	67.30	1416	13.03	1.47	0.33	0.39	87.00	< LOQ	81.71
	**Median**	7800	2.62	167.64	0.63	0.20	79.56	1566	15.34	1.78	0.35	0.12	91.48	NA	103.24
	**SD**	3958	1.05	93.13	0.36	0.42	55.99	652.66	10.15	0.91	0.16	0.53	68.11	NA	56.63
BRAND B (*n* = 6)	**Min–max**	2043**–**3741	3.82**–**5.95	27.38**–**30.22	2.26**–**2.88	26.60**–**33.08	17.71**–**37.86	3882**–**4774	0.35**–**0.41	0.12**–**0.29	< LOQ	< LOQ**–**1.11	4.42**–**5.26	< LOQ	1.99**–**2.48
	**Mean**	3244.83	4.69	28.69	2.55	29.17	23.70	4465.83	0.39	0.21	< LOQ	0.27	5.02	< LOQ	2.22
	**Median**	3458	4.63	28.48	2.50	28.42	21.93	4524	0.39	0.22	NA	0.10	5.13	NA	2.21
	**SD**	654	0.72	1.23	0.22	2.42	7.64	333.55	0.02	0.07	NA	0.41	0.31	NA	0.18
BRAND C (*n* = 7)	**Min–max**	9792**–**21,200	4.98**–**12.53	< LOQ**–**212	0.28**–**0.83	0.62**–**2.64	1.02**–**3.70	19.58**–**111.10	< LOQ**–**0.39	< LOQ**–**2.74	< LOQ	< LOQ**–**1.05	5.99**–**21.98	< LOQ	0.52**–**1.61
	**Mean**	12,603	7.44	93.84	0.45	1.38	1.88	78.93	0.23	0.94	< LOQ	0.64	10.54	< LOQ	0.89
	**Median**	10,910	7.21	57.39	0.43	1.32	1.62	81.34	0.25	0.62	NA	0.78	7.78	NA	0.63
	**SD**	4218	2.57	83.94	0.19	0.66	0.85	28.72	0.09	0.96	NA	0.38	5.64	NA	0.48
BRAND D (*n* = 5)	**Min–max**	116**–**1392	0.23**–**0.34	< LOQ**–**0.66	< LOQ	< LOQ	< LOQ	< LOQ	< LOQ	0.29**–**0.62	< LOQ	< LOQ	< LOQ**–**1.08	< LOQ	< LOQ
	**Mean**	931.62	0.30	0.45	< LOQ	< LOQ	< LOQ	< LOQ	< LOQ	0.42	< LOQ	< LOQ	0.89	< LOQ	< LOQ
	**Median**	1224	0.30	0.45	NA	NA	NA	NA	NA	0.38	NA	NA	1.01	NA	NA
	**SD**	553	0.04	0.21	NA	NA	NA	NA	NA	0.13	NA	NA	0.25	NA	NA
BRAND E (*n* = 6)	**Min–max**	9867**–**37,970	4.68**–**6.72	15.08**–**32.20	0.53**–**0.65	2.49**–**4.04	0.94**–**134.60	5409**–**6227	0.26**–**0.51	< LOQ	< LOQ	< LOQ	12.92**–**50.79	< LOQ	3.39**–**4.76
	**Mean**	25,117	5.58	24.34	0.61	2.98	43.33	5762	0.32	< LOQ	< LOQ	< LOQ	32.89	< LOQ	4.28
	**Median**	26,410	5.39	24.99	0.62	2.83	10.07	5724	0.29	NA	NA	NA	34.16	NA	4.57
	**SD**	12,100	0.89	6.97	0.05	0.57	59.71	282.34	0.10	NA	NA	NA	15.70	NA	0.60
BRAND F (*n* = 6)	**Min–max**	8232**–**12,450	3.08**–**5.49	18.56**–**29.09	0.24**–**0.35	1.44**–**2.57	0.61**–**19.59	2319**–**3783	0.30**–**0.50	< LOQ	< LOQ	< LOQ**–**1.23	4.93**–**10.46	< LOQ	1.59**–**2.71
	**Mean**	9744	4.06	22.91	0.26	2.01	5.79	2965	0.387	< LOQ	< LOQ	0.43	7.29	< LOQ	2.09
	**Median**	9467	3.81	22.41	0.25	1.99	1.87	2967	0.40	NA	NA	0.10	7.71	NA	2.01
	**SD**	1500	0.88	3.45	0.04	0.51	7.67	527.22	0.07	NA	NA	0.52	2.10	NA	0.40
BRAND G (*n* = 6)	**Min–max**	1106**–**4318	1.32**–**3.28	11.27**–**17.83	0.13**–**0.17	0.84**–**3.99	0.45**–**28.49	2019**–**3284	< LOQ**–**0.24	< LOQ	< LOQ	< LOQ**–**1.33	5.00**–**6.58	< LOQ	1.11**–**1.85
	**Mean**	2065	1.91	13.89	0.15	2.04	5.71	2688.83	0.15	< LOQ	< LOQ	0.46	5.85	< LOQ	1.58
	**Median**	1595	1.54	12.94	0.15	1.85	1.12	2822	0.14	NA	NA	0.24	5.97	NA	1.6
	**SD**	1247	0.75	2.53	0.02	1.27	11.18	449.52	0.05	NA	NA	0.50	0.58	NA	0.28
BRAND H (*n* = 6)	**Min–max**	16,570**–**28,520	6.14**–**7.33	33.16**–**59.66	0.40**–**0.61	1.53**–**2.23	0.75**–**40.15	123.70**–**143.80	0.20**–**0.33	< LOQ	< LOQ	< LOQ	17.96**–**35.26	< LOQ	1.18**–**2.09
	**Mean**	21,385	6.54	49.47	0.45	1.72	7.47	133	0.26	< LOQ	< LOQ	< LOQ	26.40	< LOQ	1.46
	**Median**	21,150	6.39	53.91	0.42	1.57	0.98	132.95	0.25	NA	NA	NA	24.40	NA	1.31
	**SD**	4105	0.42	11.47	0.08	0.28	16.01	6.49	0.05	NA	NA	NA	7.32	NA	0.36
BRAND I (*n* = 6)	**Min–max**	7607**–**10,820	2.43**–**3.05	47.78**–**62.16	0.25**–**0.37	1.10**–**2.56	0.21**–**3.97	3.70**–**10.85	0.15**–**0.37	< LOQ**–**0.23	< LOQ**–**0.81	< LOQ	59.19**–**1103	< LOQ**–**4.92	0.87**–**1.50
	**Mean**	9456	2.78	53.92	0.31	1.77	1.24	7.07	0.23	0.12	0.26	< LOQ	294	1.32	1.20
	**Median**	9497	2.75	52.95	0.32	1.75	0.42	7.30	0.19	0.09	0.11	NA	75.02	0.43	1.23
	**SD**	1313	0.23	5.76	0.05	0.50	1.53	3.03	0.09	0.06	0.28	NA	417	1.80	0.23
BRAND J (*n* = 4)	**Min–Max**	1117**–**1126	0.33**–**0.34	2.80**–**2.85	< LOQ	0.71**–**0.77	< LOQ	4.63**–**4.88	< LOQ	< LOQ	< LOQ	< LOQ	2.39**–**2.65	< LOQ	< LOQ
	**Mean**	1121	0.33	2.82	< LOQ	0.73	< LOQ	4.75	< LOQ	< LOQ	< LOQ	< LOQ	2.48	< LOQ	< LOQ
	**Median**	1120	0.34	2.81	NA	0.73	NA	4.74	NA	NA	NA	NA	2.44	NA	NA
	**SD**	4.36	0.01	0.02	NA	0.03	NA	0.10	NA	NA	NA	NA	0.12	NA	NA

In this study, the results were considered using the lowest values of the maximum permissible limit for each element generated by the Food and Drug Administration (FDA) of the United States (US) for Cr, World Health Organization (WHO) for Co and Ni, and by Federal Office of Consumer Protection and Food Safety (BVL) for As, Cd, Sb, Hg, and Pb as reported at the previous study (Mercan et al. 2023). To our knowledge, the number of studies regarding the determination of inorganic elements in toy makeup products is quite limited in the literature; therefore, comparisons were conducted by using available child or adult cosmetics.

A total of 10 different brands of toy makeup (*n* = 63) were examined, and the concentrations of inorganic elements under investigation were found significantly different (*p* < 0.05) among brands. Our results showed that a total of 57 toy makeup samples which account for 90.48% of all samples (*n* = 63) exceeded the maximum permissible limits for at least one toxic element, whereas 6 samples (9.52%) were observed within the permissible limits. Among brands, the highest Pb concentration was observed in brand A, specifically in the sample A-4 coded, with a concentration of 171.200 µg g^−1^ which was 85-fold higher than the permissible limit set by BVL. In a recent study, the concentration of Pb was found in the range of 0.05–12.20 µg g^−1^ for child toy makeup, which was much lower than our findings (< LOQ–171.20 µg g^−1^) (Kopru & Soylak 2024). While the highest mean concentration of Pb was obtained at 363.889 µg g^−1^ in adult cosmetic research, in the current study, it was obtained at 81.71 µg g^−1^ (Mercan et al. 2023).

Lead poisoning is the most common poisoning among pediatric intoxications and still an important health concern that affects about 890.000 preschoolers. This toxic element enters the child’s body mainly through the gastrointestinal tract via hand-to-mouth behavior or finger-sucking. Children, who have deficiencies of calcium, zinc, and iron elements as well as malnutrition, are considered at greater risk due to the competition of Pb with these divalent inorganic elements in the body. Also, children have a higher Pb absorption rate (30.00–75.00%) in comparison to adults (11.00%), which makes them more vulnerable to toxicity (J. Huang et al. 2023; Malik et al. 2022; Verstraeten et al. 2008). Exposure to Pb, even at low concentrations, can cause neurological disorders, mental retardation, hyperactivity, and emotional and behavioral abnormalities in children (Al osmaet al. 2019; CDC 2012; Hong et al. 2015; Kim et al. 2016; Roy et al. 2009; Sanders et al. 2009).

Mercury, another toxic element, is generally associated with impairment of neurological development (e.g., low verbal intelligence quotient (IQ), mental retardation, and loss of memory), growth disorders, damaged cerebellum, insomnia, and digestive and immune system problems in children (X. Huang et al. 2014; Liu et al. 2010; Myers et al. 2009; Oken and Bellingerb 2008; WHO 2017). In this study, only 2 samples (BRAND I-1 and I-3) exceeded the limit for Hg; however, the rest of the concentrations of samples were observed < LOQ. The concentration of Hg ranged from 0.004 to 0.90 µg g^−1^ in Kopru et al.’s paper, which was lower than those of the current study (< LOQ–4.92 µg g^−1^) (Kopru & Soylak 2024). Moreover, the maximum mean concentration of Hg was found fairly higher in this study when compared to the results of Mercan et al.’s paper (0.45 µg g^−1^) (Mercan et al. 2023).

Another toxic element, As, is considered the most toxic and carcinogenic threat, often added in trace amounts to cosmetic products due to its pigment properties. Moreover, it is also known to induce genotoxic and cytotoxic damage (Benbrahim-Tallaa et al. 2005; Gentry et al. 2010; Shankar et al. 2014; Tapio & Grosche 2006). According to our results, the highest As concentration was detected as 27.68 µg g^−1^ in the BRAND A-4 coded sample, which was remarkably higher than those of a previous study (0.84 µg g^−1^) (Kopru & Soylak 2024). On the other hand, the mean concentration of As in adult cosmetic products was observed compatible with this study. Only nine samples (14.29%) exceeded the permissible limits, which were predominantly found in BRAND A.

Cadmium exposure leads to numerous adverse health impacts on children, for instance, osteoporosis, pediatric cancer, renal damage, cardiovascular diseases, immune system abnormalities, and mental development (Chunhabundit 2016; Sigel et al. 2013; Kippler et al. 2012; Schoeters et al. 2006; Sherief et al. 2015; Takaki et al. 2004; Zheng et al. 2023). The current study showed that 2 in 10 brands were above the permissible limit set for Cd element; however, other brands were found < LOQ. According to the recent paper, the concentrations of Cd were determined with a concentration range < 0.01–0.25 µg g^−1^, which is consistent with our study in toy makeup samples (Kopru & Soylak 2024). Similarly, trace amounts of Cd levels were reported in adult cosmetics as well (Mercan et al. 2023).

Antimony is generally found in drinking water, rice, leafy vegetables, meat, and poultry and is associated with attention deficit hyperactivity disorder in children (Lee et al. 2018; Wu et al. 2011). Based on our results, Sb exceeded the safe limit in 12 samples (19.05%), especially in BRAND C. Al-Saleh et. al. reported the mean concentration of Sb as 0.052 µg g^−1^ which was lesser than the present study (Al-Saleh & Al-Enazi 2011).

The most prevalent and limit-exceeding elements in toy makeup products were found as Cr, Co, and Ni at ratios of 73.02% (*n* = 46), 55.56% (*n* = 35), and 77.78% (*n* = 49), respectively. These elements are also well-known as causes of contact dermatitis in children and adults (Brandão & Gontijo 2012; Mercan et al. 2022). Based on the literature, the risk of skin contact allergy increases when the concentrations of Co, Cr, and Ni are above the limit of 5 mg kg^−1^ in cosmetic samples, indicating it is unsafe to use (Basketter et al. 1993, 2003). In the current study, Cr concentrations exceeded this limit in 17 samples, whereas Ni concentrations exceeded all products found in BRAND B (*n* = 6). However, Co concentrations did not exceed this limit and were considered safe in terms of skin allergy risk. The exposure to Cr has been related to cancer, gastrointestinal, hematological, and immune system abnormalities, as well as kidney and liver dysfunction. Additionally, Cr has been found to cause skin ulcers, increased skin sensitivity, and respiratory complications (asthma, nasal itching, and pneumonia) (Halasova et al. 2009; Neghab et al. 2015; Saha et al. 2011).

Unlike 8 toxic elements, other inorganic elements (Al, Mn, Cu, Zn, Se, and Ba) were also detected in most toy makeup samples with varying concentrations. Since there is no maximum permissible limit exists yet for Al, Mn, Cu, Zn, Se, and Ba elements, the concentrations of these elements could not compare with any limits. The primary exposure route of these elements is oral, but to a lesser extent, dermal absorption is seen as well (Salles et al. 2023). However, these elements are frequently involved in toy products as shown in this study as well and continuous exposure to high concentrations of these elements could cause adverse health effects.

The non-essential element, Ba, is extensively found in the environment (e.g., water, food, soil, toy, and cosmetics), and after exposure to this element, it exerts negative effects on gastrointestinal, cardiovascular, and respiratory systems (Al osman et al. 2019; ATSDR 2007). It was revealed that the majority of samples contained Ba, and the highest Ba concentrations were found in BRAND A and BRAND I with concentrations 222.24 and 294.31 µg g^−1^, respectively.

Aluminum is also widely found in the environment (e.g., water, soil, and air) as a result of anthropogenic activities and ingested by the body via the dermal or oral route after consuming and/or using contaminated products such as food, cosmetics, antiperspirant, and sunscreen. The excessive use of these products can cause accumulation of Al in the body, especially in the brain, and further cause neurodegenerative diseases (e.g., Autism and Alzheimer’s disease) (Alasfar & Isaifan 2021; Mohamed et al. 2015; Sanajou et al. 2021). The Al element is also suspected to be an endocrine and skeletal system disruptor in adults and children (ATSDR 2008). Besides, children, aged 3–6 years, have the highest dietary aluminum intake (Tietz et al. 2019). The current study demonstrated that all of the toy products contained Al element including wide range concentrations between 931.620 and 25,117.833 µg g^−1^; however, the concentration range of Al was reported as 80.40–822.80 µg g^−1^ in a study, which is much lower results than the present study (Kopru & Soylak 2024).

Manganese is one of the essential elements required for enzyme functioning and cellular respiration for various organ systems, especially for the brain (M. R. Khan et al. 2021). However, at high concentrations, Mn is a neurotoxicant associated with neurological disorders in both children and adults (Shaffer et al. 2023). There has been increasing evidence that Mn may adversely affect the neurodevelopment of children (Neal & Guilarte 2013).

Since Mn absorption rates are higher in infants and children than in adults, these age groups are more susceptible to Mn poisoning as well, similar to Pb. Hence, co-exposure to these elements can increase the negative health impacts, especially on children (Andersen et al. 1999; Dorner et al. 1989; Henn et al. 2010; K. Khan et al. 2012; Menezes-Filho et al. 2011; Neal & Guilarte 2013). This study also determined Mn concentrations in toy makeup samples and obtained the highest concentration in BRAND A with 151.24 µg g^−1^, which was significantly greater than Kopru et al.’s study (19.20 µg g^−1^) (Kopru & Soylak 2024). Contrary to child cosmetics, the concentration of Mn was found much higher in adult cosmetics which was determined as 15.57–3017.86 µg g^−1^ (Mercan et al. 2023).

Copper is the main component of certain enzymes in the body and is required for metabolic functions. Additionally, insufficient intake of Cu may lead to various diseases such as defects in connective tissue, anemia, and reduced white blood cell production. However, over-exposure to this essential metal can pose negative effects on the kidneys and liver (Vella & Attard 2019). The results showed that Cu concentrations varied from < LOQ to 164.68 µg g^−1^ in toy makeup samples, which was remarkably higher than the maximum concentration presented in a published study (9.46 µg g^−1^) (Kopru & Soylak 2024).

Zinc also plays a crucial role in vital functions in the body; however, it also poses a threat to human health when exposed in high amounts, especially to gastrointestinal, reproductive, and excretion systems (Agnew & Slesinger 2020; Materna & Nieradko-Iwanicka 2020). It was obtained that the highest mean concentration of Zn was found in BRAND E with a concentration of 5723.50 µg g^−1^, whereas the maximum concentration of Zn was found as 13.30 µg g^−1^ in a previous study (Kopru & Soylak 2024).

Although Se is considered an essential inorganic element, excessive exposure to this element also can lead to some diseases such as selenosis. In this study, Se concentrations were obtained lower than other inorganic elements through toy makeup samples. Based on our results, the highest mean concentration of Se was observed as 1.47 µg g^−1^ in BRAND A, whereas the maximum concentration of Se was found to be 11.52 µg g^−1^ in a recent adult cosmetic study (Mercan et al. 2023).

Since there is limited data in the literature regarding toxic element contents of toy makeup products, results could not compare sufficient amounts of papers. Corazza et al. (2009) found the Ni, Cr, and Co concentrations in the ranges of 1.40–320.00, 1.61–3620.00, 0.47–12.50 mg kg^−1^, respectively, in toy eye shadow samples (Corazza et al. 2009), and these ranges are higher than those of this study. Another study related to the inorganic profile of toy makeup products indicated the highest concentrations of 72.30 and 54.50 µg g^−1^ for Cr and Pb, respectively (Rastogi 1992), whereas this study found the highest concentrations of 12.530 and 171.200 µg g^−1^ for Co and Pb, respectively.

### Health risk assessment

The mean SED values were calculated and compared with TDI values as presented in Table 2 (Van Engelen et al. 2008). Although Cr, Co, Ni, As, Cd, Sb, and Hg did not exceed TDI values, Al and Pb exceeded the TDI values at ratios 54.55% (*n* = 6) and 9.09% (*n* = 8), respectively. According to these results, there was a health concern about Al and Pb concentrations in toy makeup samples.

**Table 2:** Systemic exposure dosage (SED) and tolerable daily intake (TDI) values of inorganic elements in samples (mg kg^−1^ d^−1^)

	Al	Cr	Mn	Co	Ni	Cu	Zn	As	Se	Cd	Sb	Ba	Hg	Pb
SED (mean value)														
BRAND A (*n* = 11)	**1.12E + 00**	4.18E − 04	2.72E − 02	1.11E − 04	6.53E − 05	1.21E − 02	2.55E − 01	2.35E − 03	2.65E − 04	5.86E − 05	7.00E − 05	1.57E − 02	NA	**1.47E − 02**
BRAND B (*n* = 6)	5.84E − 01	8.44E − 04	5.16E − 03	4.58E − 04	5.25E − 03	4.27E − 03	8.04E − 01	6.93E − 05	3.76E − 05	NA	4.83E − 05	9.04E − 04	NA	4.00E − 04
BRAND C (*n* = 7)	**2.27E + 00**	1.34E − 03	1.69E − 02	8.01E − 05	2.49E − 04	3.39E − 04	1.42E − 02	4.13E − 05	1.69E − 04	NA	1.15E − 04	1.90E − 03	NA	1.61E − 04
BRAND D (*n* = 5)	1.68E − 01	5.35E − 05	8.05E − 05	NA	NA	NA	NA	NA	7.48E − 05	NA	NA	1.60E − 04	NA	–-
BRAND E (*n* = 6)	**4.52E + 00**	1.00E − 03	4.38E − 03	1.09E − 04	5.36E − 04	7.80E − 03	1.04E + 00	5.82E − 05	NA	NA	NA	5.92E − 03	NA	7.71E − 04
BRAND F (*n* = 6)	**1.75E + 00**	7.31E − 04	4.12E − 03	4.73E − 05	3.61E − 04	1.04E − 03	5.34E − 01	6.97E − 05	NA	NA	7.75E − 05	1.31E − 03	NA	3.76E − 04
BRAND G (*n* = 6)	3.72E − 01	3.44E − 04	2.50E − 03	2.61E − 05	3.68E − 04	1.03E − 03	4.84E − 01	2.77E − 05	NA	NA	8.30E − 05	1.05E − 03	NA	2.84E − 04
BRAND H (*n* = 6)	**3.85E + 00**	1.18E − 03	8.90E − 03	8.12E − 05	3.09E − 04	1.35E − 03	2.39E − 02	4.61E − 05	NA	NA	NA	4.75E − 03	NA	2.63E − 04
BRAND I (*n* = 6)	**1.70E + 00**	5.01E − 04	9.71E − 03	5.64E − 05	3.19E − 04	2.22E − 04	1.27E − 03	4.06E − 05	2.16E − 05	4.66E − 05	NA	5.30E − 02	2.37E − 04	2.16E − 04
BRAND J (*n* = 4)	2.02E − 01	6.01E − 05	5.08E − 04	NA	1.32E − 04	–-	8.55E − 04	NA	NA	NA	NA	4.46E − 04	NA	NA
TDI value														
	7.50E − 01	5.00E − 03	–-	1.40E − 03	1.00E − 02	–-	–-	1.00E − 03	–-	5.00E − 04	6.00E − 03	–-	2.00E − 03	3.60E − 03

*NA*, not applicable.

Also, the worst-case scenario (95th percentiles) was computed and shown in Table 3 for toy makeup. The results of the worst-case scenario were compared with TDI values given in Table 2. According to the worst-case exposure scenario results, 7 out of 10 brands (BRAND A, C, E, F, G, H, and I) exceeded the TDI levels for Al. Also, the worst-case exposure scenario results exceeded the TDI levels in BRAND A for both Pb and As as the prominent toxic elements, while the rest of the worst-case exposure results of inorganic elements (Cr, Co, Ni, Cd, Sb, and Hg) were found within the TDI limits.

**Table 3:** The worst-case scenario (95th percentiles) values of the fourteen considered inorganic elements for children’s toy makeups (mg kg^−1^ d^−1^)

	Al	Cr	Mn	Co	Ni	Cu	Zn	As	Se	Cd	Sb	Ba	Hg	Pb
The worst-case scenario (95th percentiles)														
BRAND A (*n* = 11)	1.81 E + 00	6.00E − 04	4.00 E − 02	2.00E − 04	NA	2.00E − 02	3.70 E − 01	4.00E − 03	4.00E − 04	1.00E − 04	NA	2.60E − 02	NA	2.00E − 02
BRAND B (*n* = 6)	6.70 E − 01	1.00E − 03	1.00 E − 02	5.00E − 04	6.00E − 03	7.00E − 03	8.60 E − 01	1.00E − 04	1.00E − 04	NA	2.00E − 04	9.00E − 04	NA	4.00E − 04
BRAND C (*n* = 7)	3.82 E + 00	2.00E − 03	4.00 E − 02	1.00E − 04	5.00E − 04	1.00E − 03	2.00 E − 02	1.00E − 04	5.00E − 04	NA	2.00E − 04	4.00E − 03	NA	3.00E − 04
BRAND D (*n* = 5)	2.50 E − 01	1.00E − 04	1.00 E − 04	NA	1.00E − 04	3.00E − 04	2.00 E − 04	NA	1.00E − 04	NA	NA	2.00E − 04	NA	NA
BRAND E (*n* = 6)	6.83 E + 00	1.00E − 03	6.00 E − 03	1.00E − 04	7.00E − 04	2.00E − 02	1.12 E + 00	1.00E − 04	NA	NA	NA	9.00E − 03	NA	9.00E − 04
BRAND F (*n* = 6)	2.24 E + 00	1.00E − 03	5.00 E − 03	1.00E − 04	5.00E − 04	4.00E − 03	6.80 E − 01	1.00E − 04	NA	NA	2.00E − 04	2.00E − 03	NA	5.00E − 04
BRAND G (*n* = 6)	7.80 E − 01	6.00E − 04	3.00 E − 03	1.00E − 04	7.00E − 04	5.00E − 03	5.90 E − 01	1.00E − 04	NA	NA	2.00E − 04	1.00E − 03	NA	3.00E − 04
BRAND H (*n* = 6)	5.13 E + 00	1.00E − 03	1.10 E − 02	1.00E − 04	4.00E − 04	7.00E − 03	3.00 E − 02	1.00E − 04	NA	NA	NA	6.00E − 03	NA	4.00E − 04
BRAND I (*n* = 6)	1.95 E + 00	5.00E − 04	1.10 E − 02	1.00E − 04	5.00E − 04	7.00E − 04	2.00 E − 03	1.00E − 04	1.00E − 04	1.00E − 04	NA	2.00E − 01	9.00E − 04	3.00E − 04
BRAND J (*n* = 4)	2.00 E − 01	1.00E − 04	5.00 E − 04	NA	1.00E − 04	NA	9.00 E − 04	NA	NA	NA	NA	5.00E − 04	NA	NA

*NA*, not applicable

The risk assessments of toy makeup products were calculated based on the MoS approach as well and considered as if the MoS value is greater than 150, it indicates safe to use for < 5 aged children. The results of estimated mean MoS values based on Eq. 1 for the toy makeup products at 100% bio-accessibility are presented in Table 4. The MoS values of Cr, Ni, Se, and Ba were found to be ˃150, which indicated safe. On the other hand, the MoS values of Al, Mn, Co (only brand B), Zn, As, Cd, Sb, Hg, Cu (only 1 sample in brand A), and Pb (only 1 sample in brand A) were obtained < 150, which considered unsafe to use. Among inorganic elements, Mn (*n* = 53), Al (*n* = 42), and Zn (*n* = 32) were the most MoS limit exceeding elements. The A, B, C, D, E, F, and G brands exceeded the proposed limit for MoS, but Hg exceeded the limit only in BRAND I. Only two brands (A and I) were shown to exceed the MoS limit for Cd; however, the rest of the brands were could not calculated. As a result, the most potentially risky brand was observed as brand A, whereas brands D and J were found within safe limits. Moreover, although no maximum permissible limits exist for Al, Mn, and Zn, the MoS-based health risk assessment showed that these elements have the most unsafe MoS values among other elements.

**Table 4:** Mean values of margin of safety (MoS) for inorganic elements in toy makeup samples (assuming 100% bioaccessibility)

	Al	Cr	Mn	Co	Ni	Cu	Zn	As	Se	Cd	Sb	Ba	**Hg**	**Pb**
BRAND A (*n* = 11)	**89.53**	4662.96	**3.53**	270.60	30,639.82	330.20	**117.69**	**12.79**	1887.66	**21.33**	**85.77**	89,395.45	NA	271.98
BRAND B (*n* = 6)	171.21	2309.55	**18.59**	**65.48**	380.93	937.52	**37.30**	432.71	13,301.41	NA	**124.22**	1,548,227.28	NA	9994.25
BRAND C (*n* = 7)	**44.08**	1455.81	**5.68**	374.41	8047.36	11,799.71	2111.46	726.90	2957.33	NA	**52.13**	738,279.81	NA	24,900.84
BRAND D (*n* = 5)	596.33	36,426.81	1192.61	NA	NA	NA	NA	NA	6683.78	NA	NA	8,754,815.15	NA	NA
BRAND E (*n* = 6)	**22.12**	1943.025	**21.92**	275.18	3733.36	512.90	**28.93**	515.73	NA	NA	NA	236,502.47	NA	5189.28
BRAND F (*n* = 6)	**57.02**	2667.32	**23.28**	634.52	5532.96	3835.94	**56.21**	430.29	NA	NA	**77.40**	1,066,739.82	NA	10,643.68
BRAND G (*n* = 6)	269.03	5666.96	**38.41**	1148.11	5435.96	3895.33	**61.99**	1084.60	NA	NA	**72.33**	1,329,913.56	NA	14,109.35
BRAND H (*n* = 6)	**25.98**	1655.50	**10.78**	369.28	6475.01	2973.67	1253.13	651.47	NA	NA	NA	294,594.20	NA	15,220.70
BRAND I (*n* = 6)	**58.75**	3891.28	**9.89**	531.92	6278.65	17,993.70	23,560.46	739.10	23,148.15	**26.85**	NA	26,426.86	**126.42**	18,531.39
BRAND J (*n* = 4)	495.81	32,435.13	189.08	NA	15,189.49	NA	35,108.05	NA	NA	NA	NA	3,139,048.64	NA	NA

*NA*, not applicable.

**Fig. 2:**
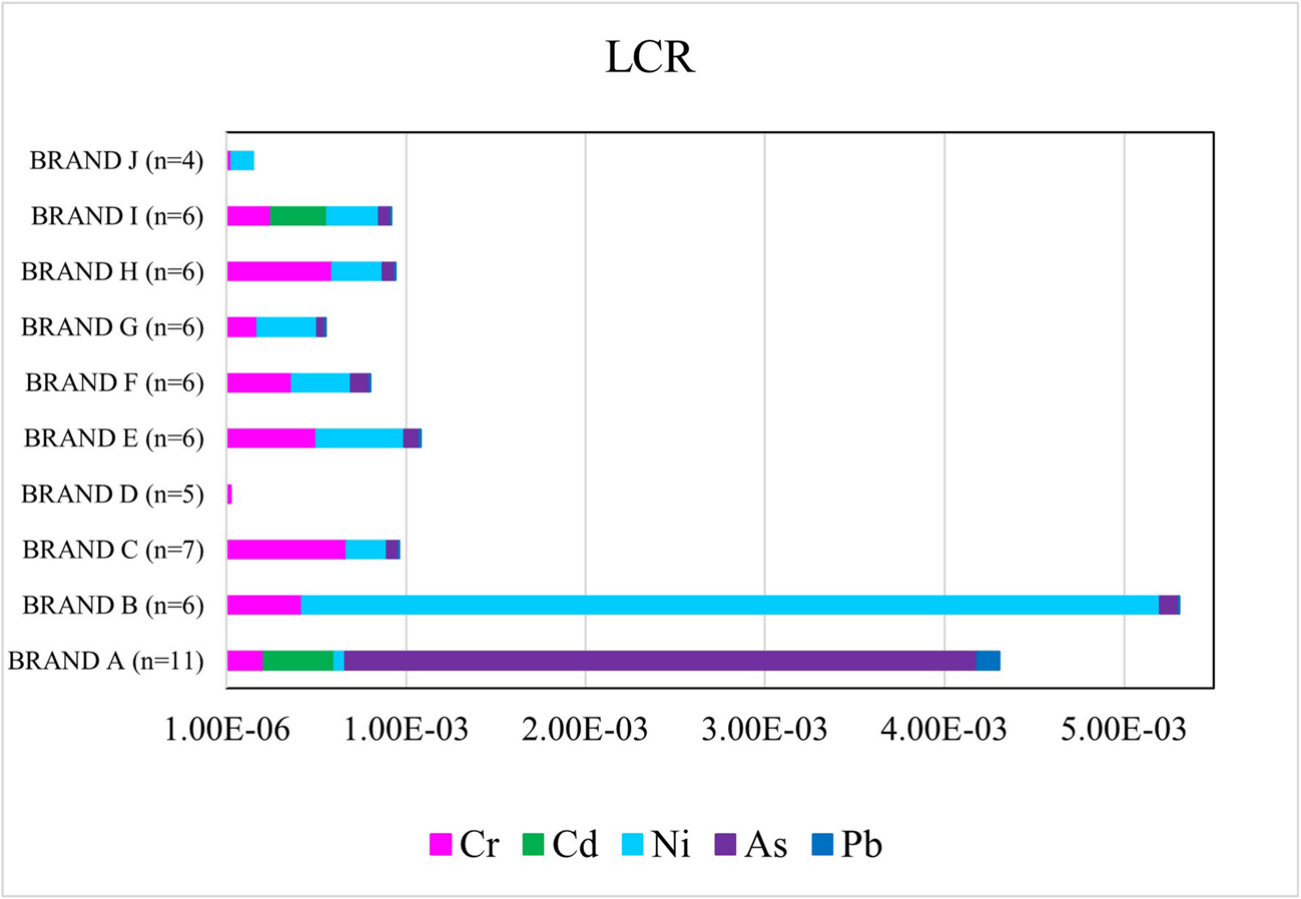
The comparison of mean lifetime cancer risk (LCR) values of ten brands

Since Cr, Pb, Ni, As, and Cd are listed as carcinogenic elements by the International Agency for Research on Cancer (IARC) (IARC 2022), LCR was calculated to estimate the potential cancer risk of each toy makeup products (Table S2). The LCR values were found to be ˃ 10^−6^ for Cr (100.00%, *n* = 63), Ni (79.37%, *n* = 50), As (85.71%, *n* = 54), Cd (18.87%, *n* = 10), and Pb (77.78%, *n* = 49), which indicate unsafe products. The BRAND D and BRAND J did not exceed the LCR value for Cd, Pb, and As and were considered safe in toy makeup products. According to this study, the most potential cancer risk was found in the descending order as Cr ˃ As ˃ Ni ˃ Pb ˃ Cd in samples as shown in Fig. 2.

**Fig. 3:**
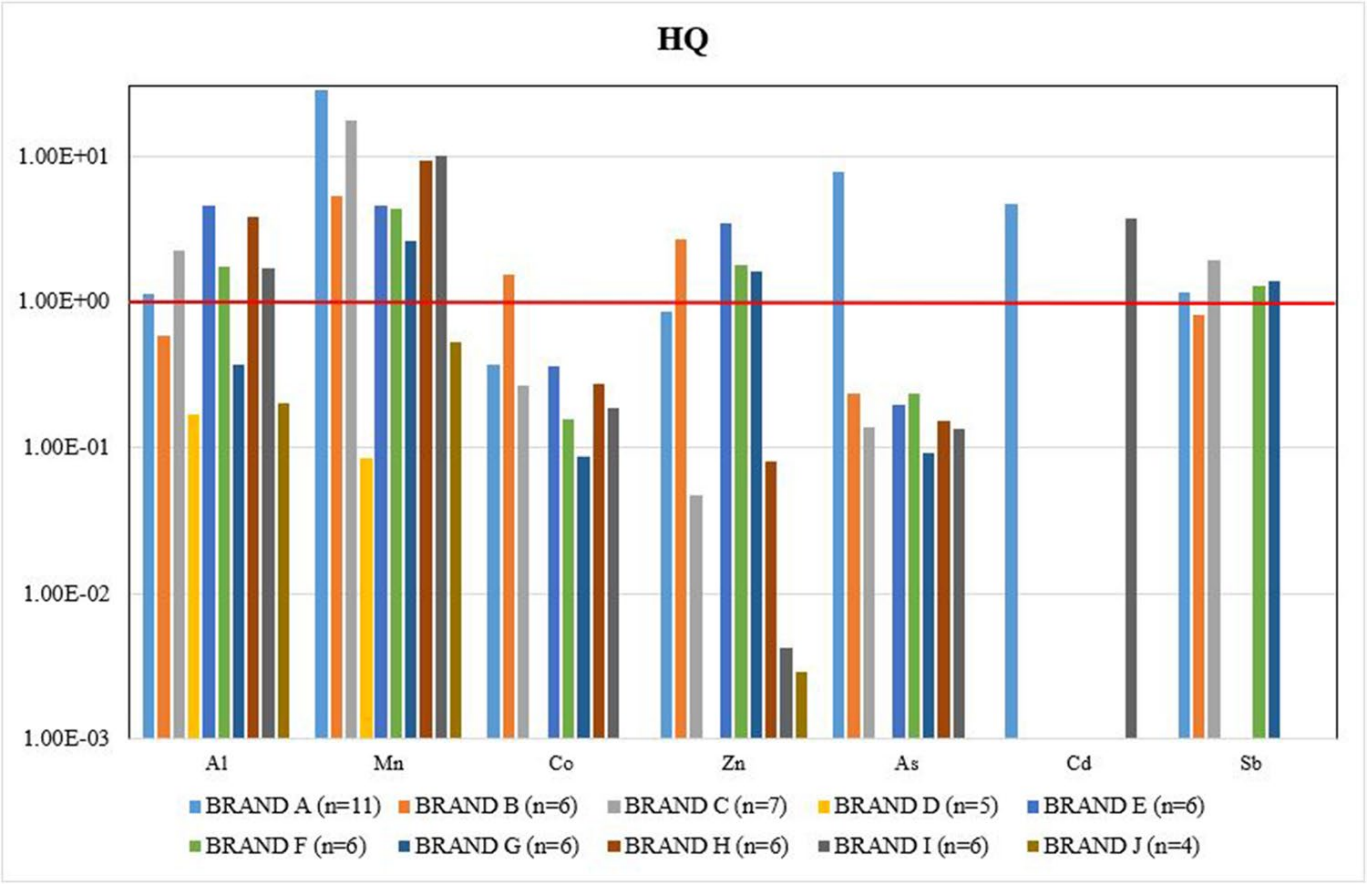
The comparison of mean hazard quotient (HQ) values of ten brands

The HQ values (Fig. 3) exceeded the safe limit (≥ 1) in BRAND A, for As and Cd, whereas in BRAND I, only Cd exceeded this limit. For BRAND B, the Co element was found above the limit set for HQ, but in BRAND I, only Hg exceeded the limit. Nickel, Cr, Cu, Se, Ba, and Pb were found within the safe limit ( < 1). While the BRAND D and BRAND J were found within the safe limit for all elements, which indicated no apparent risk for human health in terms of both HQ and HI values, the rest of the brands (A, B, C, E, F, G, H, and I) were found above the limit set for HI, which indicated unsafe products to use.

The contamination of children’s toy makeup products with significant amounts of hazardous elements has become an important health concern due to the risk of the natural behaviors of children (hand to mouth, hand in mouth, nail-biting, finger sucking, or pica) (Guney & Zagury 2012). There are many toxic elements-related intoxication cases after playing with toys of children up to date (Njati & Maguta 2019). This study showed for the first time that toy makeup products were contaminated with significant amounts of inorganic elements which could further cause mild to severe effects on a child’s health. Furthermore, co-exposure to these elements could contribute to the severity of health effects, and multi-route exposure (dermal and oral) to these inorganic elements increases the rate of toxicity in children.

Children are already exposed to inorganic elements via water, soil, and air as well as food, sunscreen, vaccine, milk, and toothpaste as adults (Abedi et al. 2020; Sarker et al. 2022). Another source of exposure is toy products as shown in this study, and additionally, contamination of these products could increase the body load of such elements and further cause various adverse effects.

### Allergenic fragrance analysis of toy makeup products

**Fig. 4:**
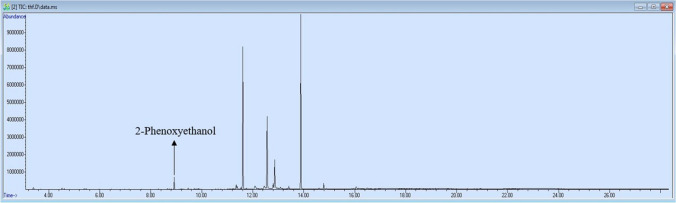
Total ion chromatogram of 2-phenoxyethanol

The toy makeup products were analyzed with a GC–MS system in scan mode in terms of allergen fragrance ingredients. Based on our result, only 2-phenoxyethanol was qualitatively determined in toy makeup samples as an allergen (Fig. 4). This allergen is mostly used as an anti-microbial agent in cosmetic products (Scognamiglio et al. 2012). There are some case reports as well as review studies in the literature including 2-phenoxyethanol-related diseases such as contact dermatitis, acute toxicity, mucous membrane irritation, phototoxicity, and photoallergy (Birnie & English 2006; Dréno et al. 2019; Kolodziej et al. 2022; Scognamiglio et al. 2012).

Previous studies found different allergens in cosmetics by using different extraction methods such as solid phase dispersion-pressurized liquid extraction (Lamas et al. 2010; Sanchez-Prado et al. 2011), liquid–liquid extraction (Bawazir et al. 2023; Lu et al. 2021), headspace (Desmedt et al. 2015), and solid phase microextraction (Chen et al. 2023; Lamas et al. 2009; Masuck et al. 2010; Riboni et al. 2021) followed by GC–MS. Limonene, lilial, and geraniol are the most detected allergen fragrances which are already listed in SCCS (Dréno et al. 2019). To our knowledge, however, there is limited study regarding allergens in toy makeup (Rastogi et al. 1999). On the other hand, there is no allergen fragrance listed in SCCS was found in this study with a quick liquid–liquid extraction method (SCCS 2012). For further investigation of organic contaminants in toy products, substance-specific extraction methods should be developed and also other potential allergens (e.g., coloring agents, paraben, and bisphenol) should be analyzed.

## Conclusion

This study investigated the concentrations of 14 inorganic elements as well as allergen fragrance content of the toy makeup products (*n* = 63) which were purchased from toy stores in Istanbul for the first time. Prominent results were obtained for toxic, non-toxic, and essential elements. Although considering limits used in this study were regulated for adults, toy makeups exceeded the maximum permissible limits set by regulatory agencies in the descending order of Ni ˃ Cr ˃ Co ˃ Pb ˃ Sb ˃ Cd ˃ As ˃ Hg. The skin sensitization risk for Cr and Ni was observed at ratios of 26.98% and 9.52%, respectively.

The systemic exposure dosage values of Al and Pb were found to over tolerable daily intake values remarkably. The margin of safety values for Al, Mn, Co, Zn, As, Cd, Sb, and Hg were found < 150, which indicates unsafe products used by children. The lifetime cancer risk of toy makeups for Cr, Ni, Cd, Pb, and As exceeded the permissible limit at least for one element, which considers potential cancer risk. Considering the hazard quotient, 80% of all products exceeded the limit (≥ 1) at least for one inorganic element. It can be obtained from this study that long-term exposure to inorganic contaminants in these products could lead to harmful effects on children such as cancer and dermal sensitivity. These results not only showed the risk of exposure for dermal but also oral route due to the natural behaviors of children such as hand-to-mouth, hand-in-mouth, nail-biting, finger-sucking, or pica. Manufacturers should become more aware of the potentially toxic effects of inorganic contaminants in children’s toys and should reduce or remove all toxic inorganic elements during their manufacturing processes. All materials (e.g., chemicals and raw materials) and equipment that are involved in the process of toy cosmetic products should be tested before production. Authorities should increase enforcement for the production of toxic-free toy makeup. Regular controls should be tightened on children’s products, and if products fail to meet quality control standards, financial sanctions should be applied to the manufacturer. More appropriate policies should be enforced to protect children from unsafe products. Public awareness, notably among parents, should be raised by related authorities regarding the toxic effects of inorganic elements on children’s health and maximum permissible limits should be set for children-specific cosmetic products in terms of inorganic elements.

In conclusion, most of the toy makeup samples collected in the market were found unsafe to use and pose a possible threat to children’s health.

### Supplementary Information

Below is the link to the electronic supplementary material.

Supplementary file1 (DOCX 40 KB)
