# Supplementary material for: Experimental study for inorganic and organic profiling of toy makeup products: Estimating the potential threat to child health
Source: Environ Sci Pollut Res Int. 2024 May 2;31(23):33975–92. doi: 10.1007/s11356-024-33362-2 (PMC11136717; doi:10.1007/s11356-024-33362-2)
Supplement: Supplementary file 1 — Supplementary file1 (DOCX 40 KB) [file 11356_2024_33362_MOESM1_ESM.docx]

**Experimental Study for Inorganic and Organic Profiling of Toy Makeup Products: Estimating The Potential Threat to Child Health**

Selda Mercan*; Mihriban Dilan Kilic; Simge Zengin; Murat Yayla

* Corresponding author: mercans@iuc.edu.tr; Phone: +90 (212) 866 37 00, Fax: +90 (212)866 37 72

Istanbul University - Cerrahpasa Institute of Forensic Sciences and Legal Medicine

Department of Science, 34500, Buyukcekmece, Istanbul, Türkiye

**Table S1.** Concentrations of 8 toxic elements in 63 toy makeup (µg g^-1^)

| Sample Code | Cr | Co | Ni | As | Cd | Sb | Hg | Pb |
| --- | --- | --- | --- | --- | --- | --- | --- | --- |
| BRAND A-1 | 0.83 | 0.16 | **0.64** | 0.14 | <LOQ | **1.25** | <LOQ | 0.37 |
| BRAND A-2 | 0.65 | 0.13 | **1.55** | 0.125 | <LOQ | **1.62** | <LOQ | 1.01 |
| BRAND A-3 | 1.60 | **0.37** | <LOQ | **7.36** | **0.26** | <LOQ | <LOQ | **75.32** |
| BRAND A-4 | **3.54** | **0.66** | <LOQ | **27.68** | **0.46** | 0.26 | <LOQ | **171.20** |
| BRAND A-5 | **3.41** | **0.86** | <LOQ | **18.70** | **0.51** | 0.16 | <LOQ | **102.52** |
| BRAND A-6 | **3.13** | **1.07** | <LOQ | **22.69** | **0.35** | 0.12 | <LOQ | **110.92** |
| BRAND A-7 | **2.62** | **1.01** | <LOQ | **21.34** | **0.39** | 0.36 | <LOQ | **107.00** |
| BRAND A-8 | **2.27** | **0.57** | <LOQ | **15.34** | **0.48** | <LOQ | <LOQ | **114.04** |
| BRAND A-9 | **3.12** | **0.63** | <LOQ | **8.67** | **0.49** | <LOQ | <LOQ | **103.24** |
| BRAND A-10 | **3.02** | **1.10** | <LOQ | **21.13** | **0.32** | <LOQ | <LOQ | **112.20** |
| BRAND A-11 | 1.38 | 0.22 | <LOQ | 0.17 | <LOQ | <LOQ | <LOQ | 0.9 |
| BRAND B-1 | **4.25** | **2.50** | **28.20** | 0.40 | <LOQ | <LOQ | <LOQ | **2.48** |
| BRAND B-2 | **4.87** | **2.72** | **31.00** | 0.41 | <LOQ | <LOQ | <LOQ | **2.36** |
| BRAND B-3 | **4.60** | **2.50** | **28.63** | 0.35 | <LOQ | <LOQ | <LOQ | **2.09** |
| BRAND B-4 | **4.66** | **2.40** | **27.50** | 0.38 | <LOQ | <LOQ | <LOQ | 1.99 |
| BRAND B-5 | **5.95** | **2.88** | **33.08** | 0.38 | <LOQ | <LOQ | <LOQ | **2.25** |
| BRAND B-6 | **3.82** | **2.26** | **26.60** | 0.40 | <LOQ | **1.11** | <LOQ | **2.17** |
| BRAND C-1 | **5.73** | 0.28 | **0.62** | 0.25 | <LOQ | <LOQ | <LOQ | 1.58 |
| BRAND C-2 | **5.51** | 0.29 | **1.14** | <LOQ | <LOQ | <LOQ | <LOQ | 0.52 |
| BRAND C-3 | **7.21** | **0.43** | **1.32** | 0.271 | <LOQ | **0.87** | <LOQ | 0.60 |
| BRAND C-4 | **4.98** | **0.34** | **0.84** | 0.16 | <LOQ | **1.05** | <LOQ | 1.61 |
| BRAND C-5 | **7.95** | **0.46** | **1.42** | 0.16 | <LOQ | **0.78** | <LOQ | 0.70 |
| BRAND C-6 | **12.53** | **0.83** | **2.64** | 0.28 | <LOQ | **0.78** | <LOQ | 0.63 |
| BRAND C-7 | **8.19** | **0.49** | **1.67** | 0.39 | <LOQ | **0.80** | <LOQ | 0.61 |
| BRAND D-1 | 0.34 | <LOQ | 0.45 | <LOQ | <LOQ | <LOQ | <LOQ | <LOQ |
| BRAND D-2 | 0.29 | <LOQ | <LOQ | <LOQ | <LOQ | <LOQ | <LOQ | <LOQ |
| BRAND D-3 | 0.23 | <LOQ | <LOQ | <LOQ | <LOQ | <LOQ | <LOQ | <LOQ |
| BRAND D-4 | 0.32 | <LOQ | <LOQ | <LOQ | <LOQ | <LOQ | <LOQ | <LOQ |
| BRAND D-5 | 0.30 | <LOQ | <LOQ | <LOQ | <LOQ | <LOQ | <LOQ | <LOQ |
| BRAND E-1 | **4.82** | **0.65** | **2.61** | 0.32 | <LOQ | <LOQ | <LOQ | **4.56** |
| BRAND E-2 | **6.72** | **0.59** | **4.04** | 0.31 | <LOQ | <LOQ | <LOQ | **3.67** |
| BRAND E-3 | **4.68** | **0.59** | **2.69** | 0.26 | <LOQ | <LOQ | <LOQ | **4.58** |
| BRAND E-4 | **5.88** | **0.53** | **3.06** | 0.26 | <LOQ | <LOQ | <LOQ | **3.39** |
| BRAND E-5 | **6.44** | **0.64** | **2.96** | 0.28 | <LOQ | <LOQ | <LOQ | **4.74** |
| BRAND E-6 | **4.90** | **0.64** | **2.49** | **0.51** | <LOQ | <LOQ | <LOQ | **4.76** |
| BRAND F-1 | **3.81** | 0.239 | **1.55** | 0.40 | <LOQ | **1.23** | <LOQ | 1.90 |
| BRAND F-2 | **5.49** | **0.35** | **2.57** | 0.50 | <LOQ | <LOQ | <LOQ | **2.71** |
| BRAND F-3 | **4.72** | 0.25 | **2.27** | 0.40 | <LOQ | <LOQ | <LOQ | 1.84 |
| BRAND F-4 | **3.47** | 0.24 | **2.51** | 0.30 | <LOQ | <LOQ | <LOQ | 1.59 |
| BRAND F-5 | **3.08** | 0.240 | **1.44** | 0.39 | <LOQ | **0.95** | <LOQ | **2.36** |
| BRAND F-6 | **3.81** | 0.257 | **1.70** | 0.34 | <LOQ | <LOQ | <LOQ | **2.12** |
| BRAND G-1 | 1.49 | 0.15 | **1.01** | <LOQ | <LOQ | <LOQ | <LOQ | 1.46 |
| BRAND G-2 | 1.32 | 0.15 | **1.06** | <LOQ | <LOQ | 0.38 | <LOQ | 1.75 |
| BRAND G-3 | 1.58 | 0.13 | **2.63** | 0.18 | <LOQ | <LOQ | <LOQ | 1.85 |
| BRAND G-4 | **3.28** | 0.17 | **3.99** | 0.24 | <LOQ | <LOQ | <LOQ | 1.79 |
| BRAND G-5 | **2.30** | 0.15 | **2.73** | 0.18 | <LOQ | **1.33** | <LOQ | 1.48 |
| BRAND G-6 | 1.51 | 0.13 | **0.84** | <LOQ | <LOQ | **0.75** | <LOQ | 1.11 |
| BRAND H-1 | **6.40** | **0.40** | **1.58** | 0.25 | <LOQ | <LOQ | <LOQ | 1.18 |
| BRAND H-2 | **6.67** | **0.44** | **1.84** | 0.23 | <LOQ | <LOQ | <LOQ | 1.28 |
| BRAND H-3 | **6.38** | **0.41** | **1.53** | 0.24 | <LOQ | <LOQ | <LOQ | 1.34 |
| BRAND H-4 | **6.14** | **0.43** | **1.55** | 0.28 | <LOQ | <LOQ | <LOQ | 1.68 |
| BRAND H-5 | **6.34** | **0.41** | **1.57** | 0.20 | <LOQ | <LOQ | <LOQ | 1.20 |
| BRAND H-6 | **7.33** | **0.61** | **2.23** | 0.33 | <LOQ | <LOQ | <LOQ | **2.09** |
| BRAND I-1 | **2.78** | **0.36** | **2.56** | 0.275 | **0.30** | <LOQ | **1.27** | 1.20 |
| BRAND I-2 | **2.43** | 0.28 | **1.60** | 0.21 | <LOQ | <LOQ | <LOQ | 1.00 |
| BRAND I-3 | **3.05** | 0.25 | **1.10** | 0.374 | **0.81** | <LOQ | **4.92** | 1.38 |
| BRAND I-4 | **2.72** | **0.34** | **1.89** | 0.17 | <LOQ | <LOQ | <LOQ | 1.25 |
| BRAND I-5 | **3.02** | **0.37** | **2.01** | 0.17 | <LOQ | <LOQ | <LOQ | 0.87 |
| BRAND I-6 | **2.71** | 0.29 | **1.45** | 0.15 | <LOQ | <LOQ | <LOQ | 1.50 |
| BRAND J-1 | 0.33 | <LOQ | **0.74** | <LOQ | <LOQ | <LOQ | <LOQ | <LOQ |
| BRAND J-2 | 0.34 | <LOQ | **0.71** | <LOQ | <LOQ | <LOQ | <LOQ | <LOQ |
| BRAND J-3 | 0.33 | <LOQ | **0.77** | <LOQ | <LOQ | <LOQ | <LOQ | <LOQ |
| BRAND J-4 | 0.34 | <LOQ | **0.71** | <LOQ | <LOQ | <LOQ | <LOQ | <LOQ |

**Table S2.** The Lifetime Cancer Risk (LCR) value of 5 elements in 63 toy makeup (µg g^-1^)

| **Sample Code** | **Cr** | **Ni** | **Cd** | **Pb** | **As** |
| --- | --- | --- | --- | --- | --- |
| **BRAND A-1** | **7.44E-05** | **1.04E-04** | NA | 5.68E-07 | **1.00E-04** |
| **BRAND A-2** | **5.82E-05** | **2.55E-04** | NA | **1.55E-06** | **2.73E-04** |
| **BRAND A-3** | **1.44E-04** | NA | **3.14E-04** | **1.15E-04** | **2.03E-02** |
| **BRAND A-4** | **3.19E-04** | NA | **5.60E-04** | **2.62E-04** | **4.62E-02** |
| **BRAND A-5** | **3.07E-04** | NA | **6.13E-04** | **1.57E-04** | **2.77E-02** |
| **BRAND A-6** | **2.82E-04** | NA | **4.20E-04** | **1.70E-04** | **2.99E-02** |
| **BRAND A-7** | **2.36E-04** | NA | **4.68E-04** | **1.64E-04** | **2.89E-02** |
| **BRAND A-8** | **2.04E-04** | NA | **5.74E-04** | **1.74E-04** | **3.08E-02** |
| **BRAND A-9** | **2.81E-04** | NA | **5.89E-04** | **1.58E-04** | **2.79E-02** |
| **BRAND A-10** | **2.71E-04** | NA | **3.86E-04** | **1.72E-04** | **3.03E-02** |
| **BRAND A-11** | **1.24E-04** | NA | NA | **1.45E-06** | **2.56E-04** |
| **BRAND B-1** | **3.82E-04** | **4.62E-03** | NA | **3.79E-06** | **6.68E-04** |
| **BRAND B-2** | **4.39E-04** | **5.08E-03** | NA | **3.62E-06** | **6.38E-04** |
| **BRAND B-3** | **4.14E-04** | **4.69E-03** | NA | **3.19E-06** | **5.63E-04** |
| **BRAND B-4** | **4.19E-04** | **4.50E-03** | NA | **3.05E-06** | **5.38E-04** |
| **BRAND B-5** | **5.36E-04** | **5.42E-03** | NA | **3.45E-06** | **6.08E-04** |
| **BRAND B-6** | **3.44E-04** | **4.36E-03** | NA | **3.32E-06** | **5.86E-04** |
| **BRAND C-1** | **5.16E-04** | **1.02E-04** | NA | **2.42E-06** | **4.26E-04** |
| **BRAND C-2** | **4.96E-04** | **1.87E-04** | NA | 7.91E-07 | **1.40E-04** |
| **BRAND C-3** | **6.49E-04** | **2.16E-04** | NA | 9.13E-07 | **1.61E-04** |
| **BRAND C-4** | **4.48E-04** | **1.38E-04** | NA | **2.46E-06** | **4.34E-04** |
| **BRAND C-5** | **7.15E-04** | **2.33E-04** | NA | **1.07E-06** | **1.89E-04** |
| **BRAND C-6** | **1.13E-03** | **4.32E-04** | NA | 9.68E-07 | **1.71E-04** |
| **BRAND C-7** | **7.37E-04** | **2.74E-04** | NA | 9.38E-07 | **1.66E-04** |
| **BRAND D-1** | **3.09E-05** | **7.29E-05** | NA | NA | NA |
| **BRAND D-2** | **2.57E-05** | NA | NA | NA | NA |
| **BRAND D-3** | **2.10E-05** | NA | NA | NA | NA |
| **BRAND D-4** | **2.91E-05** | NA | NA | NA | NA |
| **BRAND D-5** | **2.72E-05** | NA | NA | NA | NA |
| **BRAND E-1** | **4.34E-04** | **4.28E-04** | NA | **6.98E-06** | **1.23E-03** |
| **BRAND E-2** | **6.05E-04** | **6.62E-04** | NA | **5.62E-06** | **9.92E-04** |
| **BRAND E-3** | **4.21E-04** | **4.41E-04** | NA | **7.00E-06** | **1.24E-03** |
| **BRAND E-4** | **5.29E-04** | **5.01E-04** | NA | **5.18E-06** | **9.14E-04** |
| **BRAND E-5** | **5.80E-04** | **4.85E-04** | NA | **7.25E-06** | **1.28E-03** |
| **BRAND E-6** | **4.41E-04** | **4.08E-04** | NA | **7.28E-06** | **1.28E-03** |
| **BRAND F-1** | **3.43E-04** | **2.54E-04** | NA | **2.91E-06** | **5.13E-04** |
| **BRAND F-2** | **4.94E-04** | **4.21E-04** | NA | **4.15E-06** | **7.33E-04** |
| **BRAND F-3** | **4.25E-04** | **3.72E-04** | NA | **2.81E-06** | **4.96E-04** |
| **BRAND F-4** | **3.12E-04** | **4.12E-04** | NA | **2.43E-06** | **4.30E-04** |
| **BRAND F-5** | **2.78E-04** | **2.35E-04** | NA | **3.62E-06** | **6.38E-04** |
| **BRAND F-6** | **3.43E-04** | **2.79E-04** | NA | **3.25E-06** | **5.73E-04** |
| **BRAND G-1** | **1.34E-04** | **1.66E-04** | NA | **2.23E-06** | **3.94E-04** |
| **BRAND G-2** | **1.19E-04** | **1.73E-04** | NA | **2.68E-06** | **4.73E-04** |
| **BRAND G-3** | **1.42E-04** | **4.31E-04** | NA | **2.84E-06** | **5.01E-04** |
| **BRAND G-4** | **2.95E-04** | **6.54E-04** | NA | **2.74E-06** | **4.83E-04** |
| **BRAND G-5** | **2.07E-04** | **4.48E-04** | NA | **2.27E-06** | **4.01E-04** |
| **BRAND G-6** | **1.36E-04** | **1.37E-04** | NA | **1.70E-06** | **3.01E-04** |
| **BRAND H-1** | **5.76E-04** | **2.58E-04** | NA | **1.81E-06** | **3.19E-04** |
| **BRAND H-2** | **6.01E-04** | **3.01E-04** | NA | **1.95E-06** | **3.44E-04** |
| **BRAND H-3** | **5.74E-04** | **2.51E-04** | NA | **2.04E-06** | **3.61E-04** |
| **BRAND H-4** | **5.53E-04** | **2.54E-04** | NA | **2.57E-06** | **4.53E-04** |
| **BRAND H-5** | **5.70E-04** | **2.57E-04** | NA | **1.84E-06** | **3.24E-04** |
| **BRAND H-6** | **6.59E-04** | **3.66E-04** | NA | **3.20E-06** | **5.65E-04** |
| **BRAND I-1** | **2.51E-04** | **4.19E-04** | **3.62E-04** | **1.84E-06** | **3.25E-04** |
| **BRAND I-2** | **2.19E-04** | **2.62E-04** | NA | **1.53E-06** | **2.71E-04** |
| **BRAND I-3** | **2.75E-04** | **1.81E-04** | **9.79E-04** | **2.11E-06** | **3.73E-04** |
| **BRAND I-4** | **2.45E-04** | **3.10E-04** | NA | **1.91E-06** | **3.37E-04** |
| **BRAND I-5** | **2.71E-04** | **3.30E-04** | NA | **1.32E-06** | **2.34E-04** |
| **BRAND I-6** | **2.44E-04** | **2.38E-04** | NA | **2.29E-06** | **4.04E-04** |
| **BRAND J-1** | **3.00E-05** | **1.22E-04** | NA | NA | NA |
| **BRAND J-2** | **3.03E-05** | **1.17E-04** | NA | NA | NA |
| **BRAND J-3** | **2.97E-05** | **1.25E-04** | NA | NA | NA |
| **BRAND J-4** | **3.02E-05** | **1.15E-04** | NA | NA | NA |

NA: not applicable
